# Distinct variation in vector competence among nine field populations of *Aedes aegypti* from a Brazilian dengue-endemic risk city

**DOI:** 10.1186/1756-3305-7-320

**Published:** 2014-07-11

**Authors:** Caroline M Gonçalves, Fabrício F Melo, Juliana MT Bezerra, Bárbara A Chaves, Breno M Silva, Luciana D Silva, José EM Pessanha, Jorge R Arias, Nágila FC Secundino, Douglas E Norris, Paulo FP Pimenta

**Affiliations:** 1 Laboratory of Medical Entomology, René Rachou Research Centre- FIOCRUZ, Belo Horizonte, Minas Gerais, Brazil; 2 Department of Biological Sciences, Federal University of Ouro Preto, Ouro Preto, Minas Gerais, Brazil; 3 Municipal Health Secretariat of Belo Horizonte, Belo Horizonte, Minas Gerais, Brazil; 4 Department of Internal Medicine, Federal University of Minas Gerais, Belo Horizonte, Minas Gerais, Brazil; 5 Medical Entomologist, Retired Consultant, Manassas, Virginia, USA; 6 Department of Molecular Microbiology and Immunology, Johns Hopkins Malaria Research Institute, Bloomberg School of Public Health, Johns Hopkins University, Baltimore, MD, USA

**Keywords:** *Dengue virus* (DENV), *Aedes aegypti*, Field population, Infection rate, Vector competence, Disseminated infection rate

## Abstract

**Background:**

In Brazil, dengue epidemics erupt sporadically throughout the country and it is unclear if outbreaks may initiate a sustainable transmission cycle. There are few studies evaluating the ability of Brazilian *Aedes aegypti* populations to transmit dengue virus (DENV). The aim of this study was to compare DENV susceptibility of field-captured *Ae. aegypti* populations from nine distinct geographic areas of the city of Belo Horizonte in 2009 and 2011. Infection Rate (IR), Vector Competence (VC) and Disseminated Infection Rate (DIR) were determined.

**Methods:**

*Aedes aegypti* eggs from each region were collected and reared separately in an insectary. Adult females were experimentally infected with DENV-2 and the virus was detected by qPCR in body and head samples. Data were analyzed with the Statistical Package for the Social Sciences version 17.

**Results:**

IR varied from 40.0% to 82.5% in 2009 and 60.0% to 100.0% in 2011. VC ranged from 25.0% to 77.5% in 2009 and 25.0% to 80.0% in 2011. DIR oscillated from 68.7% to 100.0% in 2009 and 38.4% to 86.8 in 2011. When the results were evaluated by a logistic model using IR as covariate, North, Barreiro, South-Central and Venda Nova showed the strongest association in 2009. In 2011, a similar association was observed for South-Central, Venda Nova, West and Northeast regions. Using VC as covariate, South-Central and Venda Nova showed the most relevant association in 2009. In 2011, South-Central, Venda Nova and Barreiro presented the greatest revelation associations. When DIR data were analyzed by logistic regression models, Pampulha, South-Central, Venda Nova, West, Northeast and East (2009) as well as South-Central, Venda Nova and West (2011) were the districts showing the strongest associations.

**Conclusions:**

We conclude that *Ae. aegypti* populations from Belo Horizonte exhibit wide variation in vector competence to transmit dengue. Therefore, vector control strategies should be adapted to the available data for each region. Further analysis should be conducted to better understand the reasons for this large variability in vector competence and how these parameters correlate with epidemiological findings in subsequent years.

## Background

Dengue is a tropical disease and the most important mosquito-vectored viral infection of humans. There are approximately 50 million people infected with dengue each year and about 500,000 of these individuals are hospitalized with dengue hemorrhagic fever, mainly in the tropical and sub-tropical countries of Southeast Asia, the Pacific and the Americas. Remarkably, despite efforts aimed at control, the annual average number of dengue cases reported to The World Health Organization (WHO) has increased dramatically in recent years. Moreover, a geographic expansion of active dengue transmission has been observed over this same time period [1,2]. In 2013, countries in the Americas reported more than 2.3 million cases of dengue, with 37,692 cases of severe dengue and 1,280 deaths, resulting in a mortality rate of about 0.05%. Brazil has reported the majority of the dengue cases during the last two decades [3].

*Dengue virus* (DENV) is a positive-strand RNA virus (*Flavivirus*; *Flaviviridae*). The DENV complex consists of four closely related but antigenically distinct serotypes (DENV-1, −2, −3 and −4). They are transmitted by several mosquito species of the genus *Aedes* (*Stegomyia*)*.* For all DENV serotypes *Ae. aegypti* is the primary vector. The co-circulation of the 4 serotypes increases the risk of severe forms of dengue. Six years ago, DENV-4 was detected in Manaus, the capital city of the state of Amazonas infecting patients with an acute febrile illness [4]. All four DENV serotypes are currently circulating or co-circulating in different areas of Brazil including Belo Horizonte, the city where this study was conducted. In recent years, dengue outbreaks have led to many atypical cases, including myocarditis, hepatitis, meningoencephalitis and acute kidney failure, and fatality rates have also increased [4].

The mosquito *Ae. aegypti* is distributed in subtropical and tropical areas of the globe and is now endemic in many countries. In the Americas, *Ae. aegypti* is predominantly found in urban and semi-urban areas of 35 nations and 9 territories [3], although, in recent years, its presence has also been reported in rural areas [5,6]. Most Brazilian states are infested with *Ae. aegypti* and consequently at risk of dengue transmission [4]. Due to the importance of *Ae. aegypti* as a vector of dengue and yellow fever virus, this mosquito species has been the subject of numerous studies focusing on population genetics and vector competence [7].

*Aedes aegypti* becomes infected when the mosquito bites and acquires an infected blood meal from a DENV-infected human, the primary host of the virus. The mosquito infection depends on factors such as DENV virulence, physical barriers and innate immunity that can confer resistance or susceptibility of an *Ae. aegypti* population. Effectively, this intrinsic permissiveness of a vector to an infection, for example, replication and transmission of the virus to a new human host, is related to a number of anatomical barriers. The vector susceptibility will depend on completion of the DENV life cycle by crossing physical barriers, which include target organs such as the midgut, hemocoel and salivary gland [8-10]. Infectious mosquito bites after this extrinsic incubation period (EIP) can result in an infection for humans [11,12].

The transmission of DENV by *Ae. aegypti* in any geographic area depends on many factors, several of which are well defined. These include extrinsic features related to the environment and intrinsic factors associated with the virus/vector interaction including: (a) the proportion of individual mosquitoes that can be infected in a population considering presence of the virus in the insect body, which is defined as infection rate (IR); (b) the proportion of mosquitoes in which DENV can complete its life cycle (i.e., practically, virus present in the head reaching the salivary glands), which is defined as vector competence (VC) [13]; and (c) the vector’s ability to transmit DENV which is more precisely related to the disseminated infection rate (DIR), which is the proportion of DENV-infected mosquito heads (VC) in an infected population with virus dispersed in the body (IR).

Overall, the epidemiological pattern of dengue disease in tropical countries is a complex phenomenon. In Brazil, dengue epidemics are still diffusing throughout the country and outbreaks may or may not initiate a permanent and sustainable transmission cycle. Despite the importance of dengue as a disease that impacts millions of people, there are only a few studies evaluating the ability of Brazilian *Ae. aegypti* populations to transmit the virus. A better understanding of the relationship between DENV and mosquito vector is critical to the development of new and effective methodologies targeting the vector for control.

The goal of this work was to compare DENV susceptibility of field-captured *Ae aegypti* populations in the city of Belo Horizonte in two years, 2009 and 2011. The city has approximately 2.3 million inhabitants and has recorded thousands of endemic cases of dengue during the last two decades, with more than 80,000 cases reported in 2013. This study focused on intrinsic factors that affect the ability of the vector to transmit DENV by comparing the IR, VC and DIR of *Ae. aegypti* populations from nine distinct geographic areas of the city. The data presented here should be taken into account in future studies aimed at understanding dengue transmission dynamics in a large urban setting and in the development of effective strategies for vector control.

## Methods

### Study area

The research was performed in the city of Belo Horizonte, capital of Minas Gerais, located at latitude 19°49′01″, longitude 43°57′21″ and an altitude of 858 m above sea level. The city occupies an area of 332 km^2^, with a population of 2,375,151 inhabitants and population density of 7,167 inhabitants/km^2^ according to the census carried out by the Brazilian Institute of Geography and Statistics in 2010 [14]. The city has a tropical climate with temperatures ranging from 18°C to 23°C and annual average of 21°C. The relative humidity remains near 65% and the average annual rainfall is approximately 1,500 mm, being more frequent from October to March [15]. The city is divided into nine administrative regions: Pampulha, Barreiros, South-Central, Venda Nova, West, North, Northwest, East and Northeast. In the present study we collected mosquitoes from all nine regions.

### Mosquito collections

*Aedes aegypti* eggs were collected in 2009 and 2011. The collections were conducted in all districts of Belo Horizonte city. Eggs were collected with ovitraps in collaboration with the field-agents of the city’s Secretary of Health, which is responsible for detecting, monitoring and controlling mosquito populations. Larvae from field-collected eggs were hatched in aninsectary at a temperature of 28°C and relative humidity of 80%. The adults from each region were reared in separate cages and infection experiments were performed with 3–5 day old female mosquitoes.

### Virus

The dengue virus serotype 2 used in all assays was a Brazilian strain isolated from a hemorrhagic fever patient in 1991, provided by the Molecular Microbiology and Virology Laboratory of the Ezequiel Dias Foundation located in Belo Horizonte. Prior to the experiments, frozen virus samples were amplified in cultures of C6/36 cells with Leibowitz-15 medium and supplements (20 μg/mL Gentamicin, 5 μg/mL Amphotericin B, 200U/mL Penicillin plus 2% inactivated Fetal Bovine Serum). Virus titration followed the TCID50 method [16]. The mean viral titer used in the infection experiments was 5 × 10^5^ TCID_50_/mL.

### Experimental infection

Two hundred *Ae. aegypti* G_0_ females from each of the nine districts were infected via a glass feeding device containing blood and DENV-2. Briefly, the feeders were covered with a skin of young *Gallus domesticus*. A mixture comprising 2/3 of blood mouse (*Mus musculus*) and 1/3 of C6/36 cell suspension infected with DENV-2 was added to the feeders and offered to the mosquitoes as described elsewhere [17]. One hour after feeding, only fully engorged females were separated in cages and maintained with 10% glucose solution *ad libitum* until the 14^th^ day after experimental infection, the time necessary for completion of the EIP.

### Dissection and DENV-RNA extraction

A total of 40 infected females were randomly removed from each of the nine experimentally infected groups. They were carefully dissected under a stereoscope and their bodies and heads (always with attached salivary glands) placed in separate vials. Viral RNA was extracted from the two samples of each mosquito using the QIAamp viral RNA mini kit (Qiagen) according to the manufacturer’s instructions.

### Detection of DENV by real-time (qPCR)

DENV-2 detection in the body and head of *Ae. Aegypti* was facilitated using qpcr. The reaction was performed in an ABI Prism 7500 Fast Real Time PCR machine (Applied Biosystems) using the Power SYBR® Green RNA-to-Ct 1-step detection system (Applied Biosystems). The 3′Non-coding region primers, B1-forward (5′-AGGACYAGAGGTTAGAGGAGA-3′) and B2- reverse (5′-CGYTCTGTGCCTGGAWTGAT-3′) were used [18]. All analyses were performed in duplicate with standardized samples (to provide standard curves), positive control and negative control. The results were obtained using a standard curve and analyzing the melting curve for the specificity of the amplified products (~78.6°C) and to approximately CT 35, as outlined in The Minimum Information for Publication of Quantitative Real-Time PCR Experiments (MIQE) [19].

### Evaluation of infection rate, vector competence and disseminated infection rate

The Infection Rate (IR) was calculated as the proportion (percentage) of all experimentally infected mosquitoes (n = 40) in which DENV was detected (whole body). In contrast, the Vector Competence (VC) was calculated as the proportion of all experimentally infected mosquitoes (n = 40) for which DENV was detected in the head/salivary glands. The analysis of vector competence was performed according to Bennett *et al.* (2002) [19]. Conceptually, the Disseminated Infection Rate (DIR) is the proportion of DENV-infected mosquito heads (VC) of all infected mosquitoes with virus (IR) in the body (DIR = VC/IR) [19].

### Statistical analysis

Data were analyzed with the Statistical Package for the Social Sciences (SPSS) version 17 (SPSS Inc., Chicago, IL, USA). Differences of IR, VC and DIR among the vector populations were evaluated using the two-tailed chi-square or Fisher’s tests. Comparison of these parameters among the two years of study was performed using the two-tailed Students *t* test (the Kolmogorov–Smirnov goodness-of-fit was used to assess the normality of the data). *P* values ≤ 0.05 were considered significant. The association of each district covariate t with IR, VC and DIR (dependent variables) was tested by univariate analysis. All the variables with a *P*-value of 0.25 or less were included in the full logistic regression model. Odds ratio (OR) and 95% confidence interval (CI) were used as an estimate of the risk. The Hosmer–Lemeshow goodness-of-fit test was used to evaluate the fit of the models [20].

## Results

Prior to the experimental DENV-infections, sub-samples of eggs from all *Ae. aegypti* populations were screened by qRT-PCR to confirm they were negative for natural dengue virus infection. This scrutiny of possible natural infection of the mosquitoes was necessary since this study was developed with adult female mosquitoes derived from field-collected eggs from endemic areas.

### Characterization of the IR, VC and DIR of the belo horizonte city

**Figure 1:**
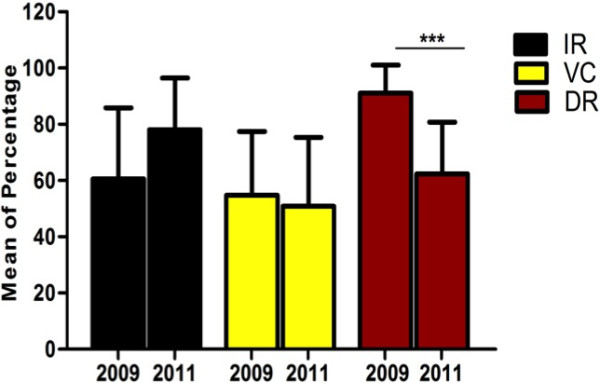
**The proportion of the analyzed characters of the DENV infection (IR, VC and DIR) is represented by the mean of the percentage found in the 9 districts during the years 2009 and 2011, which characterizes data from the entire Belo Horizonte city.** *** Notice that there is only significance comparing the DIR between the two years.

Analysis across all nine districts of Belo Horizonte from the two collections, revealed dissimilarities in the characteristics related to experimental DENV-infection of the *Ae. aegypti* mosquitoes. The infection rates (IR) varied from 40.0% to 82.5% (60.6 ± 8.4) in 2009 and 60.0% to 100.0% (mean = 78.1 ± 6.1) in 2011. The vector competence (VC), infection of mosquito heads and salivary glands, ranged from 25.0% to 77.5% (mean = 54.7 ± 7.6) in 2009 and 25.0% to 80.0% (mean = 50.8 ± 8.2) in 2011. The disseminated infection rates (DIR) oscillated from 68.7% to 100.0% (mean = 91.1 ± 3.3) in 2009 and 38.4% to 86.8 (62.4 ± 6.1) in 2011. Despite these differences between the two collection years, the differences were only statistically significant for the DIR (p = 0.008) (Figure 1).

### Infection rates (proportion of DENV detected in the body related to the total number of blood-fed mosquitoes)

In 2009, the highest IR (82.5%) was detected for *Ae. aegypti* populations from four regional districts (North, Barreiro, South-Central and Venda Nova). The lowest IR in 2009 (25.0%) was found in Pampulha. Comparative statistical analyses revealed that the IRs were significantly higher (p ≤ 10^3^) in North, Barreiro, South-Central, Venda Nova and West than in those observed in Pampulha, Northeast, East and Northwest districts. In 2011, the highest IRs were considerably higher with an IR of 100% calculated for Venda Nova, 97.5% for South-Central, and 95.0% calculated for both the West and Northeast districts. The North and Barreiro districts had similar IRs (65.0%), and the lowest IR (55.0%) was calculated for Northwest. Comparative evaluation of the IR data showed that they were significantly higher (p ≤ 0.003) in South-Central, Venda Nova, West and Northeast districts compared to the North, Barreiro, Pampulha, East and Northwest districts. No other significant differences were observed (Table 1).

**Table 1:** Details of the IR, VC and DIR of the 9 regional districts

^**1**^**Districts**	^**1**^**Infection rates (IR)**		^**2**^**Vector competence (VC)**		^**3**^**Disseminated infection rate (DIR)**	
	**2009**	**2011**	**2009**	**2011**	**2009**	**2011**
**North**	33/40 (82.5%)	26/40 (65%)	29/40 (72.5%)	14/40 (35%)	29/33 (87.8%)	14/16 (53.8%)
**Barreiro**	33/40 (82.5%)	26/40 (65%)	28/40 (70%)	10/40 (25%)	28/33 (84.8%)	10/26 (38.4%)
**Pampulha**	10/40 (25%)	24/40 (60%)	10/40 (25%)	17/40 (42.5%)	10/10 (100%)	17/24 (70.8%)
**Central South**	33/40 (82.5%)	39/40 (97.5%)	31/40 (77.5%)	32/40 (80%)	31/33 (93.9%)	32/39 (82%)
**Venda Nova**	33/40 (82.5%)	40/40 (100%)	30/40 (75%)	32/40 (80%)	30/33 (90.9%)	32/40 (80%)
**West**	31/40 (77.5%)	38/40 (95%)	29/40 (72.5%)	33/40 (82.5%)	29/31 (93.5%)	33/38 (86.8%)
**Northeast**	13/40 (32.5%)	38/40 (95%)	13/40 (32.5%)	23/40 (57.5%)	13/13 (100%)	23/38 (60.5%)
**East**	16/40 (40%)	28/40 (70%)	16/40 (40%)	11/40 (27.5%)	16/16 (100%)	11/28 (39.2%)
**Northwest**	16/40 (40%)	22/40 (55%)	11/40 (27.5%)	11/40 (27.5%)	11/16 (68.7%)	11/22 (50%)

^1^Infection rate (IR) is the proportion of DENV-infected mosquitos (incidence of virus in the body) of the total mosquito sample (n = 40). ^2^The vector competence (VC) is the proportion of DENV-infected mosquito heads (assuming the presence of virus in the salivary gland) of the total mosquito sample (n = 40). ^3^The disseminated infection rate (DIR) is the proportion of DENV-infected mosquito heads (VC) of an infected population with virus in the body (IR).

### Vector competence (proportion of DENV in the head/salivary glands of total of blood-fed mosquitoes)

For the 2009 *Ae. aegypti* populations, the highest VC was calculated for the South-Central district (77.5%) and the lowest for Pampulha district (25.0%). Comparatively, the analysis of all regional districts illustrated significantly higher VC rates (p ≤ 10^−3^) in North, Barreiro, South-Central, Venda Nova and West districts than in Pampulha, Northwest, East and Northwest districts (Table 1). In 2011, the highest VC rates were higher than those determined for 2009. In 2011 the highest VC was calculated for the mosquito population from the West district (82.5%). The next highest rate was reported for populations from the South-Central and Venda Nova districts (80.0%), and the lowest VC rate was from mosquitoes from the Barreiro district (25.0%). Comparatively, there were significantly higher VC (p ≤ 10^−3^) in South-Central, Venda Nova and West than in those observed in North, Barreiro, Pampulha, East and Northwest. Moreover, the VC rate in the Northeast region was significantly higher (p ≤ 0.009) than in Pampulha, Barreiro, East and Northwest. No other significant differences were observed (Table 1).

### Disseminated infection rate (proportion of DENV in the head/salivary glands related to the total number of mosquitoes with DENV in the body**)**

**Figure 2:**
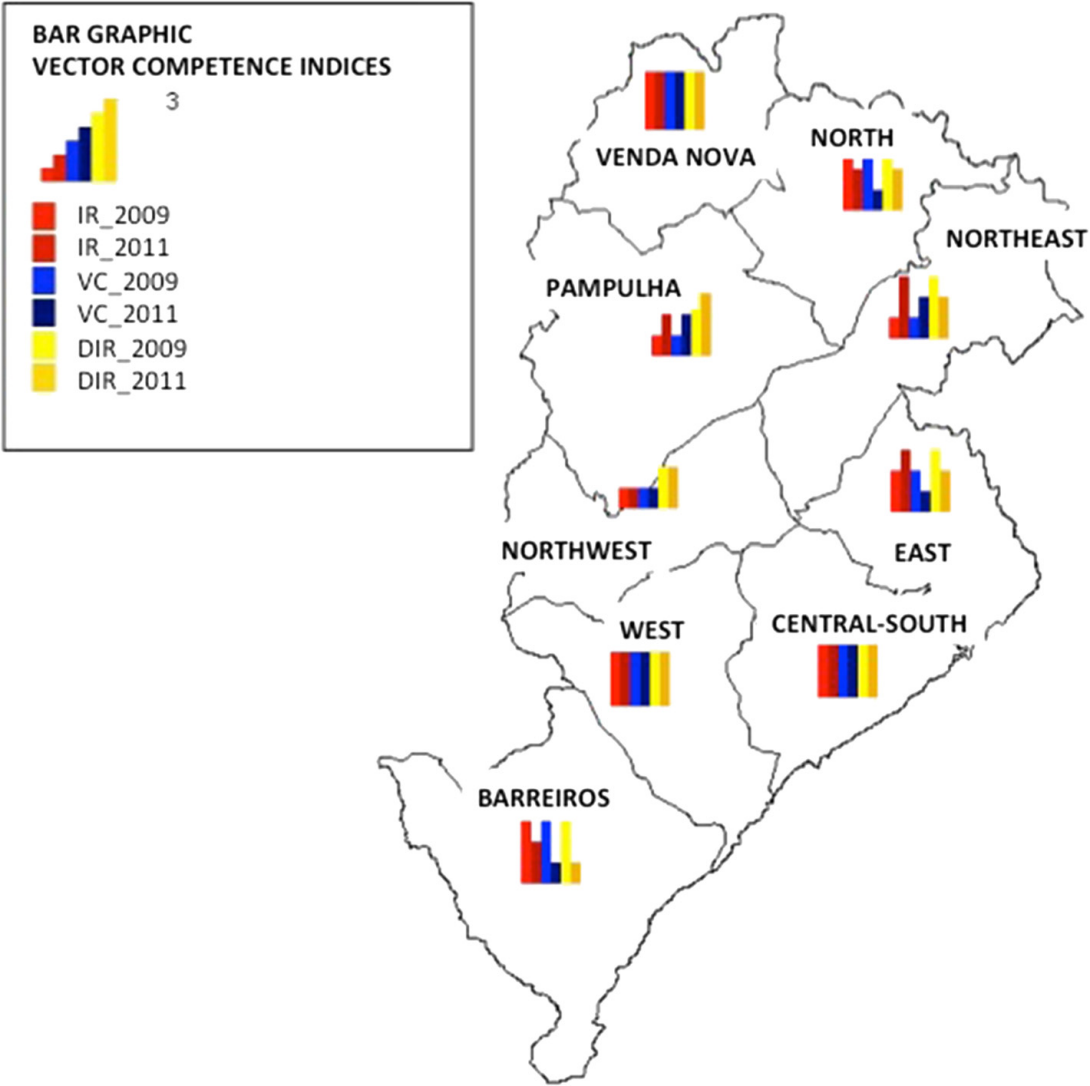
**Schematic map of the city of Belo Horizonte showing graphics of the vector competence of the *Ae. aegypti *populations in the years 2009 and 2011.** The city of Belo Horizonte is divided in 9 regional districts.

In 2009, the DIR was very similar among the regional districts, varying from 84.7% to 100%, except for the Northwest region for which a significantly lower DIR was calculated (68.7%) (p = 0.003). The mosquito populations of North, Pampulha, South-Central, Venda Nova, West, Northwest and East presented DIRs significantly higher than Barreiro and Northwest districts (p ≤ 0.009). However, this profile was not observed in 2011 when the DIR varied from 38.4% (Barreiro) to 86.8% (West). The mosquitoes of North, Pampulha, South-Central, Venda Nova, West, and Northeast presented DIRs significantly higher than Barreiro and East districts (p ≤ 0.009) (Table 1). Figure 2 summarizes the IRs, VCs and DIRs found in all regional districts of Belo Horizonte showing plots directly superposed over the city map.

### Statistical analysis of the IR, VC and DIR of the *Ae. aegypti* populations

Results were evaluated in a logistic model having the IR as covariate. In 2009, North, Barreiro, South-Central and Venda Nova presented the strongest associations. In 2011, the same was observed for South-Central, Venda Nova, West and Northeast regions. However, considering VC as covariate, the results demonstrated the most relevant association for South-Central and Venda Nova districts in 2009. In 2011, South-Central, Venda Nova and Barreiro regions were the ones showing the strongest associations. The Hosmer-Lemeshow test showed good fit of all models. Analysis of DIR data using logistic regression models indicated that Pampulha, South-Central, Venda Nova, West, Northeast and East (2009) as well as South-Central, Venda Nova and West (2011) were the districts with the strongest associations. The Hosmer-Lemeshow test showed good fit of all models.

## Discussion

Understanding the relationship between DENV and its vector, *Ae. aegypti,* is critical for epidemiological reasons. Government health authorities fight against the vector as the only available tool for the control of dengue disease. There are no drugs or vaccines available to cure or prevent dengue in humans at this time. The presence and the density of vectors determine the intensity of the necessary vector control actions. However, an important fact must be considered: the mosquito populations vary in their permissiveness for *flavivirus* development [7,21,22]. This phenomenon is known as vector competence, which is related to the barriers encountered by the virus from its entry in the mosquito to its release in the saliva during blood feeding [6]. With this knowledge, control efforts can target mosquito populations with greater competence to transmit dengue and present higher risk for specific regional areas.

Our results, in general, illustrate that although infection rates in some *Ae. aegypti* populations were high, a significant proportion of individuals did not develop DENV infection of the heads/salivary glands by the end of the EIP. Consequently, mosquitoes with DENV in the thorax and abdomen, but not in the heads/salivary glands, were not competent vectors and would not be able to transmit viruses by bite to new vertebrate hosts such as humans. To complicate matters, this trait varied regionally among *Ae. aegypti* populations as well as between the two collection years in Belo Horizonte.

In 2011, whole body infections of mosquitoes from most regions were higher than in 2009, except for the North and Barreiro regions. However, this observation was not reflected in higher head/salivary gland infection rates or vector competences across the same regions in 2011 as compared to 2009. It would appear that most mosquito populations from 2011 were capable of acquiring the virus more efficiently than 2009 populations, but that their intrinsic barriers were more capable of blocking virus migration to the head/salivary glands. It was noticed that South-Central, Venda Nova and West regions consistently presented the highest infection rates in the body and head for both collection periods.

In 2009, most regions, except the Northwest, presented very high-disseminated infection rates, most above 80%. It seems that, once the mosquitoes from these regions acquired whole body infections, virus was capable of reaching the heads/salivary glands at high rates, likely making these populations effective vectors for the DENV. However, the same phenomena were not observed in 2011 when only the South-Central, Venda Nova and West regions showed similarly high dissemination rates. This might indicate that 2011 *Ae. aegypti* populations expressed more effective intrinsic barriers to virus migration from the body to the head/salivary gland. The findings also illustrate that biological phenotypes of mosquito populations, which are related to the mosquito-virus interaction, can vary significantly from region to region and year to year. This diversity may be explained by population fluctuations and gene flow among vector populations. It should be emphasized that the disseminated infection rates were very high, specifically in 2009, independent of the region from which the study population was acquired.

It is important to consider that besides DENV transmission to the human host by the vector (horizontal transmission), DENV is also maintained in the vector population vertically (transovarial transmission) [23-26]. Considering the higher IR but lower DIR in Belo Horizonte in 2011, it appears that the potential of transovarial transmission to maintain DENV in the mosquito populations was greater in *Ae. aegypti* captured in 2011 than 2009. In contrast, the mosquito population sampled in 2009 appeared to have a greater likelihood to transmit dengue by bite to humans than to offspring. Comparison of the number of dengue cases in these two years: 23, 332 cases in 2009 and only 8,529 cases in 2011 may support this hypothesis [27]. Moreover, based on data from the last two decades, the endemicity of dengue in Belo Horizonte shows that the number of cases peaks over a 3 year period, followed by a decrease for variable periods illustrating the nature of the epidemic and inter-epidemic cycles [28]. It is possible that in this region, non-permanence of dengue in humans is related to transovarial transmission of DENV within the *Ae. aegypti* population, thus the vector acts as the inter-epidemic viral reservoir. Recently, Chaves and collaborators [29] demonstrated that DENV virus could be passed by transovarial transmission through at least six generations of a population of *Ae. aegypti* originating from Belo Horizonte. An alternative hypothesis for DENV not being identified in the salivary glands is because it could not leave the midgut due to the mosquito midgut barrier, a major determinant of vector competence [19]. Further studies are necessary to better understand the role of transovarial transmission and maintenance of DENV in non-human/host cycles during inter-epidemic years.

Other studies have also shown that *Ae. aegypti* has a continuous variation in its competence to transmit flavivirus [7,19,21,22]. The first study that evaluated the vector competence of *Ae. aegypti* populations to DENV was performed by Bennett *et al*. [19]. In this study, the authors analyzed the variation in vector competence of 24 *Ae. aegypti* populations from Mexico and the United States for DENV-2 and demonstrated that these populations show significant variation in their ability to transmit DENV. Similar results were observed in this study, except over a much smaller area. We observed considerable variation in vector competence within and between populations analyzed from short spatial and temporal collections.

## Conclusions

Changes in *Ae. aegypti* susceptibility to transmit dengue as assessed in the nine populations, during the two collection periods, could be related to movement of the insect population and/or possible mating between the individuals from different regions. These findings illustrate the need for population genetic studies to determine the genetic relatedness of these populations, and to investigate the genetic polymorphisms that may be associated with vector competence. We report significant results from this study. However, further studies need to be conducted to better understand the variability in vector competence. For example, the correlation of IR, VC and DIR data with the epidemiology of dengue could result in findings that lead to more effective control measures. We conclude, therefore, that *Ae. aegypti* populations of Belo Horizonte exhibit a wide variation in vector competence to transmit dengue viruses. These data enhance the importance of developing vector control measures in accordance with the vector competence found in each region.

## Competing interests

The authors declare that they have no competing interests.

## Authors’ contributions

PFPP, BMS, NFCS, JEMP, DEN and JRA conceived and designed the experiments; CMG, JMTB and BAC: field collection and laboratory procedures; FFM, JEMP, BMS and LDS: statistical data analysis; CMG, FFM, BMS, LDS, JEMP, JAR, NFCS, DEN and PFPP data interpretation and manuscript preparation. This manuscript is a part of the PhD thesis developed by CMG and supervised by PFPP and NFCS. PFPP and NFCS are productivity fellows of the Brazilian Council for Scientific and Technological Development (CNPq). All authors read and approved the final version of this article.

## References

[B1] Guzman MG Halstead SB Artsob H Buchy P Farrar J Gubler DJ Hunsperger E Kroeger A Margolis HS Martínez E Nathan MB Pelegrino JL Simmons C Yoksan S Peeling RW Dengue: a continuing global threat Nat Rev Microbiol 2010 8 Suppl 12 7 16 10.1038/nrmicro2460 PMC4333201 21079655

[B2] World Health Organization (WHO) [http://www.wpro.who.int/mediacentre/factsheets/fs_09032012_Dengue/en/]

[B3] Pan American Health Organization [http://www.paho.org/hq/]

[B4] Figueiredo RM Naveca FG Bastos MS Melo MN Viana SS Mourão MP Costa CA Farias IP Dengue virus Type 4, Manaus, Brazil Emerg Infect Dis 2008 14 667 669 18394292 10.3201/eid1404.071185 PMC2570911

[B5] Chaturvedi UC The curse of Dengue Indian J Med Res 2006 124 467 470 17213511

[B6] Mercado-Curiel RF Black WC Munoz Mde L A dengue receptor as possible genetic marker of vector competence in *Aedes aegypti* BMC Microbiol 2008 8 118 18625079 10.1186/1471-2180-8-118 PMC2488350

[B7] Black WC IV Bennet KE Gorrochótegui-Escalante N Barillas-Mury C Fernandez-Salas I Munoz MDL Farfán-Ale JA Olson KE Beaty BJ Flavivirus susceptibility in *Aedes aegypti* Arch Med Res 2002 33 379 388 12234528 10.1016/s0188-4409(02)00373-9

[B8] Heinz FX Auer G Stiasny K Holzmann H Mandl C Guirakhoo F Kunz C The interactions of the flavivirus envelope proteins: implications for virus entry and release Arch Virol Suppl 1994 9 339 348 7913359 10.1007/978-3-7091-9326-6_34

[B9] Hung SL Lee PL Chen HW Chen LK Kao CL King CC Analysis of the steps involved in Dengue virus entry into host cells Virology 1999 257 156 167 10208929 10.1006/viro.1999.9633

[B10] Rey L Parasitologia: parasitos e doenças parasitárias do homem nas Américas e na África 2001 Rio de Janeiro: Guanabara Koogan

[B11] Watts DM Burke DS Harrison BA Whitmire RE Nisalak A Effect of temperature on the vector efficiency of *Aedes aegypti* for dengue 2 virus Am J Trop Med Hyg 1987 36 143 152 3812879 10.4269/ajtmh.1987.36.143

[B12] Schneider JR Mori A Romero-Severson J Chadee DD Severson DW Investigations of dengue-2 susceptibility and body size among *Aedes aegypti* populations Med Vet Entomol 2007 21 370 376 18092975 10.1111/j.1365-2915.2007.00699.x

[B13] Bosio CF Harrington LC Jones JW Sithiprasasna R Norris DE Scott TW Genetic structure of *Aedes aegypti* populations in Thailand using mitochondrial DNA Am J Trop Med Hyg 2005 72 434 442 15827282

[B14] Instituto Brasileiro de Geografia e Estatísitica (IBGE) Cidades: Minas Gerais: Belo Horizonte[http://www.ibge.gov.br]

[B15] Instituto Nacional de Meteorologia (INMET) [http://www.inmet.gov.br/portal/]

[B16] Campanelli ES Botelho ACC Souza KPR Secundino NFC Cecílio AB Pimenta PFP Dengue-2 virus artificial infect of brazilian colonized *Aedes aegypti* Virus Rev Res 2006 11 12 16

[B17] Reed LJ Muench H A simple method of estimating fifty percent endpoints Am J Hyg 1938 27 493 497

[B18] Leparc-Goffart I Baragatti M Temmam S Tuiskunen A Moureau G Charrel R De Lamballerie X Development and validation of real-time one-step reverse transcription-PCR for the detection and typing of dengue viruses J Clin Virol 2009 45 61 66 19345140 10.1016/j.jcv.2009.02.010

[B19] Bennett KE Olson KE Muñoz ML Fernandez-Salas I Farfan-Ale JA Higgs S Black WC Beaty BJ Variation in vector competence for dengue 2 virus among 24 collections of *Aedes aegypti* from Mexico and the United States Am J Trop Med Hyg 2002 67 85 92 12363070 10.4269/ajtmh.2002.67.85

[B20] Hosmer DW Lemeshow S Applied Logistic Regression 2000 New York: Wiley

[B21] Severson DW Knudson DL Soares MB Loftus BJ *Aedes aegypti* genomics Insect Biochem Mol Biol 2004 34 715 721 15242713 10.1016/j.ibmb.2004.03.024

[B22] Gorrochotegui-Escalante N Lozano-Fuentes S Bennett KE Molina-Cruz A Beaty BJ Black WC IV Association mapping of segregating sites in the early trypsin gene and susceptibility to dengue-2 virus in the mosquito *Aedes aegypti* Insect Biochem Mol Biol 2005 35 771 788 15894193 10.1016/j.ibmb.2005.02.015

[B23] Higgs S Beaty BJ Marquardt WC Natural cycles of vector-borne pathogens Biology of Disease Vectors 2004 2 London: Elsevier 167 186

[B24] Mourya DT Gokhale Basu A Barde PV Sapkal GN Padbidri VS Gore MM Horizontal and vertical transmission of dengue virus type 2 in highly and lowly susceptible strains of *Aedes aegypti* mosquitoes Acta Virol 2001 45 67 71 11719984

[B25] Cecílio AB Campanelli ES Souza KPR Figueiredo LB Resende MC Natural vertical transmission by *Stegomyia albopicta* as dengue vector in Brazil Braz J Biol 2009 69 123 127 19347154 10.1590/s1519-69842009000100015

[B26] Buckner EA Alto BW Lounibos LF Vertical transmission of key west dengue-1 virus by *Aedes aegypti* and *Aedes albopictus* (Diptera: Culicidae) mosquitoes from Florida J Med Entomol 2013 50 1291 1297 24843934 10.1603/me13047 PMC4031614

[B27] Prefeitura de Belo Horizonte (PBH) Regionais de Belo Horizonte [http://portalpbh.pbh.gov.br/pbh/ecp/comunidade.do?evento=portlet&pIdPlc=ecpTaxonomiaMenuPortal&app=pbh&tax=5627&lang=pt_BR&pg=5120&taxp=0&]

[B28] Prefeitura de Belo Horizonte Dengue[http://www.pbh.gov.br/smsa/bhdengue]

[B29] Chaves BA Secundino NFC Melo FF Campolina TB Gonçalves CM Bezerra JMT Campanelli ES Guedes BAM Cecílio AB Silva BM Pessanha JEM JEM Norris DE Pimenta PFP High rate of transovarial transmision of dengue virus in *Aedes aegypti* offspring associated with fluctuating vector competence suggests that the vector is also a reservoir Parasit Vectors 2014 under revision

